# Gantzer muscle. An anatomical study

**DOI:** 10.1590/1413-78522015230200955

**Published:** 2015

**Authors:** Edie Benedito Caetano, João José Sabongi, Luiz Ângelo Vieira, Maurício Ferreira Caetano, Daniel Vinhais Moraes

**Affiliations:** 1 PUC-SP, Faculdade de Medicina, Department of Orthopedics and Traumatology, Sorocaba, SP, Brazil, 1. Department of Orthopedics and Traumatology, Faculdade de Medicina da PUC-SP (Campus Sorocaba), Sorocaba, SP, Brazil

**Keywords:** Muscle, skeletal, Forearm, Forearm, Forearm, Median nerve, Cadaver

## Abstract

**OBJECTIVE:**

The relationship of Gantzer muscle to the median and anterior interosseous nerve is debated.

**METHODS:**

Ìn an anatomical study with 80 limbs from 40 cadavers the incidence, origin, insertion, nerve supply and relations of Gantzer muscle have been documented.

**RESULTS:**

The muscle was found in 54 forearms (68% of limbs) and was supplied by the anterior interosseous nerve. It arose from the deep surface of the flexor digitorum superficialis muscle, (42 limbs), coronoid process (eight limbs) and medial epicondyle (seven limbs). Its insertion was to the ulnar part of flexor pollicis longus muscle. The Gantzer muscle always lay posterior to both the median and anterior interosseous nerve.

**CONCLUSION:**

The Gantzer muscle may contribute to the median nerve and anterior interosseous nerve compression. The muscle was found in 68% of limbs and should be considered a normal anatomical pattern rather than an anatomical variation.

**Level of Evidence IV, Case Series:**

.

## INTRODUCTION

In 1813, Gantzer described an accessory muscle in the forearm, this muscle could join the flexor pollicis lungus muscle and the deep finger flexor muscle.^1^^-^ 3 However, Kaplan^4^ describes that this muscle was described by Albinus almost a century before.

The Gantzer muscle is an accessory portion of the flexor pollicis longus muscle or the flexor digitorum profundus muscle located in the forearm and it is considered an anatomical variation, its percentage of occurrence varying greatly according to the authors of the works analyzed. The relation of Gantzer muscle with the anterior interosseous nerve and the median nerve is controversial. Mangini^5^ and Hemmady *et al.* 6 state that the muscle passes posterior to the median nerve and anterior to the anterior interosseous nerve, but Dellon and Mackinnone^7^ and Al Qattan^8^ stated the Gantzer muscle is always situated posterior to the median nerve and anterior to the interosseous nerve. Ballesteros *et al.* 9 reported that the Gantzer muscle is positioned parallel at the ulnar side in relation to the anterior interosseous nerve.

The objective of this study was to analyze the incidence, origin, insertion, and innervation of the Gantzer muscle. We analyzed the anatomical and topographic relationship regarding the anterior interosseous nerve and the median nerve, verifying the possibility of Gantzer muscle being the cause of the anterior interosseous nerve and the pronator teres syndrome (median nerve compression near the elbow). The interosseous nerve compression causes weakness or paralysis of the flexor pollicis lungus, flexor digitorum profundus and square pronator muscle, and the median nerve compression causes paralysis of the muscles of the thenar region and changes in sensitivity in important discriminating area of the hand. In both situations the functional deficit is important, leading to prehension and digital pinch disability.

## MATERIALS AND METHODS

The study was approved by the Ethics Committee of Faculdade de Medicina de Sorocaba da Pontifícia Universidade Católica de São Paulo, Sorocaba, SP, Brazil.

Eighty forearms of 40 adult cadavers belonging to the discipline of Anatomy, Faculdade de Medicina de Sorocaba (PUC-SP) were dissected to perform this study, 38 cadavers were male and two were female.

Their ages ranged between 45 and 77 years old, 23 were white and 17 were non-white, 28 pieces were previously prepared with formaldehyde solution at 10% and 12 forearms were freshly dissected.

Forearm deformed by trauma, malformations and scars were excluded.

The dissection was performed through a midline incision on the forearm around the lower third of the arm; two flaps including skin and subcutaneous have been folded on the radial and ulnar sides, respectively, the same was done for the fascia of the forearm exposing, thus, all muscles.

All the muscles of the forearm were dissected, analyzing its innervation and the presence of nerve communication between the nerves of the forearm. All anatomical variations found were registered, recorded and photographed. We used a 2.5x Keeler magnifying glass (Germany) for magnification. The relationship of the Gantzer Muscle with the previous interosseous and median nerves was analyzed.

## RESULTS

**Figure 1.**
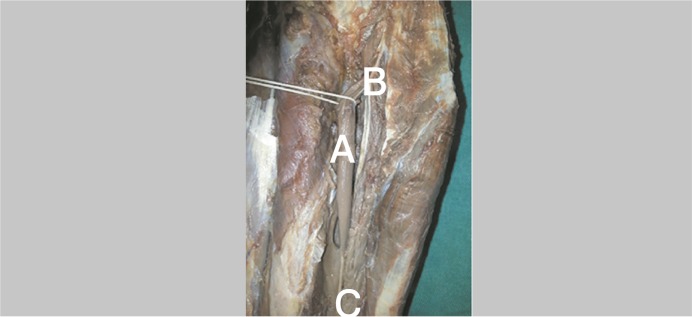
A) Gantzer muscle; B) Originating from the superficial flexor muscle; C) Inserting on the flexor pollicis lungus muscle.

The incidence of the Gantzer muscle was acknowledged in 54 of the 80 dissected forearms (68%). In 42 forearms the muscle originated from the deep portion of the flexor superficialis muscle. (Figure 1) In eight forearms it originated in the coronoid process of the ulna, and in seven from the medial epicondyle of the humerus.

**Figure 2.**
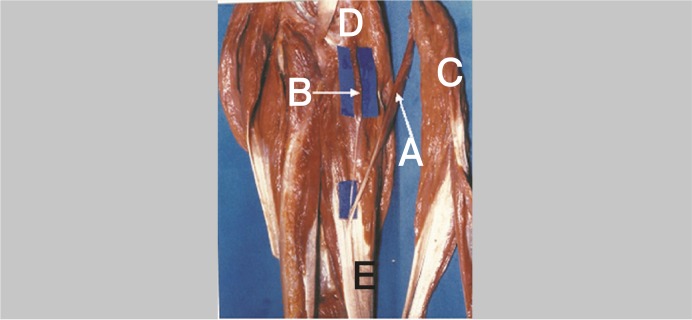
A, B) Two-headed Gantzer muscle; C) Originating from the superficial flexor muscle; D) Coronoid process of the ulna; E) Inserting on the flexor digitorum profundus.

The Gantzer muscle was inserted in the flexor pollicis lungus in 36 forearms and in the flexor digitorum profundus in 21, totaling 57 insertions in 54 forearms. In three forearms, the Gantzer muscle was duplicate. (Figure 2) In two tendons of the Gantzer muscle inserted in the proximal third of the forearm in 23 limbs, in the middle third in 20 and in the distal third in 14 limbs.

The Gantzer muscle presented fusiform shape entering through a tendon in the flexor pollicis lungus or flexor digitorum profundus.

**Figure 3.**
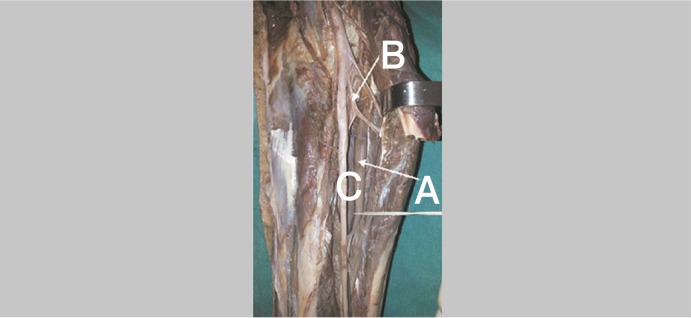
A) Gantzer muscle; B) Located posterior to the anterior interosseous nerve; C) Median nerve.

**Figure 4.**
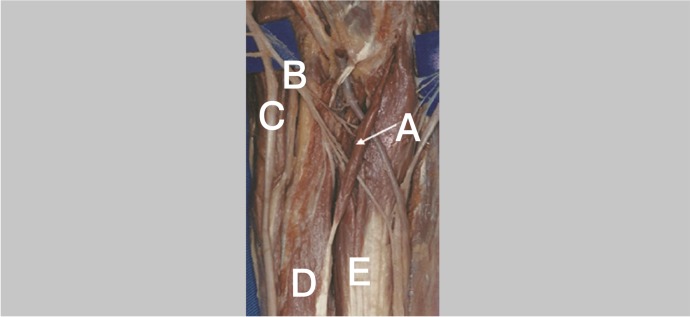
A) Gantzer muscle; B) Located anterior to the anterior interosseous nerve; C) Median nerve; D) flexor pollicis lungus muscle. E) Flexor digitorum profundus.

In all forearms we observed that the Gantzer muscle received exclusive innervation from the anterior interosseous nerve. (Figure 3) The anterior interosseous nerve was most often crossed by muscle component. (Figure 4) In only the nine pieces the interosseous nerve was crossed by the muscle tendon component.

**Figure 5.**
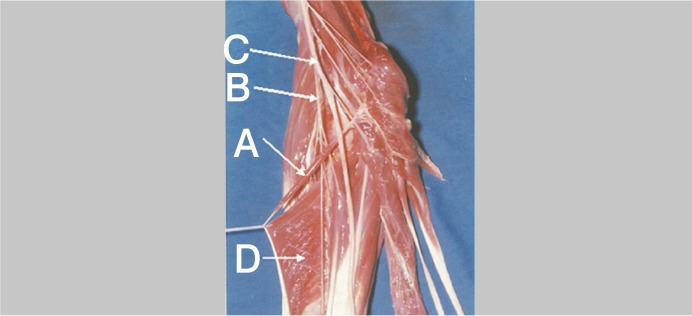
A) Gantzer muscle; B) Located anterior to the anterior interosseous nerve: C) Posterior to the median nerve; D) flexor pollicis lungus muscle.

**Figure 6.**
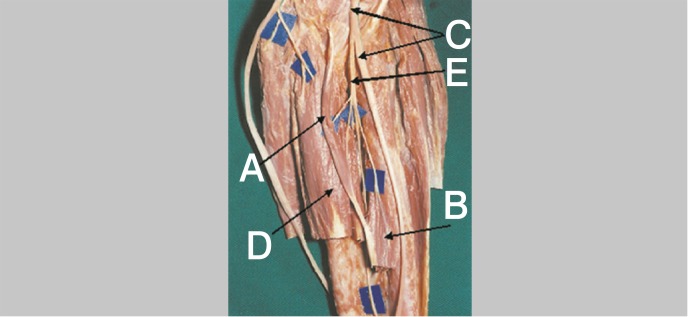
A) Gantzer muscle; B) flexor pollicis lungus muscle; C) Median nerve; D) Flexor digitorum profundus; E) Inserting on the distal third of the forearm with parallel path to the anterior interosseous nerve.

In 30 forearms the Gantzer muscle was positioned posterior to the anterior interosseous nerve, in 18 forearms it was located anterir to the nerve. (Figure 5) In six forearms the Gantzer muscle path was parallel to the anterior interosseous nerve. (Figure 6) In two pieces the Gantzer muscle was positioned anterior to the median nerve.

Of the four forearms of two female corpses, we found the Gantzer muscle in only one. We recorded the Gantzer muscle bilaterally hypertrophied in six forearms of three male patients.

## DISCUSSION

The incidence of the Gantzer muscle was recorded in 54 of the 80 dissected forearms (68%). This is close to the percentage observed by Mangini *et al.*,^5^ (71%) Hemmady *et al.*,^6^ (66.7%) and Oh *et al.* 10 (67%). Other authors have reported different results regarding the presence of this muscle: Dykes and Anson^11^ (53.3%), 54.2% Malhotra *et al.* 12, Dellon and Mackinnon^7^ (45%), Al Qattan^8^ (52%), Jones *et al.* 13 (55%), Shirali *et al.* 14 (55%), Gunnal *et al.* 15 (51.1%) and Temang *et al.* 16 (43%).

Saxena *et al.* 17 reported that during routine dissection they found the presence of Gantzer muscle bilaterally in one corpse, citing that this muscle was a very rare finding; this statement is in disagreement with the aforementioned authors, since almost all of them found the presence of muscle in more than 50% of the dissected limbs.

The important difference between our results with those of Tamang *et al*.^16^ was that of the 30 corpses dissected 10 were female, while of the 40 cadavers dissected in the present work only two were female. In the four forearms of two female corpses the presence of Gantzer muscle was noticed in only one, but we cannot say that this muscle is more common in males, although it is possible that it may occur.

We noticed that the Gantzer muscle occurred more frequently bilaterally than unilaterally, agreeing with the findings of Jones *et al*.^13^ and Hemmady *et al*.^6^

Regarding the most frequent location of Gantzer muscle origin a major disagreement stands among most authors. According to Mangini^5^ and Hemmady *et al*.,^6^ the most frequent site of Gantzer muscle origin is the medial epicondyle of the humerus. Al Qattan^8^ reports it to be the septum between the pronator and round muscles and the flexor mass of the proximal third of the forearm, while Dykes and Anson^11^ and Malhotra *et al*.^12^ suggest the medial epicondyle and coronoid process of the ulna, Oh *et al*.^10^ in the coronoid process of the ulna, and Shirali *et al*.^14^ in the flexion-pronator muscle group.

We agree with Jones *et al*.^13^ who reported the flexor digitorum superficialis muscle the site of most frequent origin. In 42 limbs the Gantzer muscle arose from the deep portion of the flexor digitorum superficialis, in eight limbs from the coronoid process of the ulna in seven in the medial epicondyle of the humerus, totaling 57 origins, because in three limbs we found duplication of the Gantzer muscle.

Regarding duplication, we recorded in three cases, Shirali *et al*.^14^ also recorded four duplications in 60 dissections, Jones *et al*.^13^ found duplication of the Gantzer muscle in two limbs, Oh *et al*.^10 ^also reported duplication in one of the limbs of 72 dissected corpses. The disagreement regarding the origin of Gantzer muscle can be explained by the way of interpretation of the dissected limbs. The flexor digitorum superficialis muscle consists of two parts, one inserted in the medial epicondyle of the humerus and the other in the coronoid process of the ulna, these two portions are joined forming an arch. Hollinshead^18^ and Caetano^19^ interpreted that in most cases the Gantzer muscle united with deep part of the flexor muscle superficialis and with it inserted into the medial epicondyle of the humerus or the coronoid process of the ulna. Other authors interpreted that the insertion occurred in the medial epicondyle or the coronoid process together with the flexor digitorum superficialis. We used a magnifying glass, which allowed us to analyze in detail the relationship between the Gantzer muscle and flexor digitorum superficialis. Jones *et al*.,^13^ who also dissected using a magnifying glass, showed results similar to ours.

Regarding the insertion, Al Qattan^8^ reported that in all dissected forearms the Gantzer muscle was inserted into the flexor pollicis longus muscle, Mangini^5^ agrees, since he considers the Gantzer muscle a continuation of the flexor pollicis lungus muscle. We registered that in 37 forearms (64 %) the Gantzer muscle was inserted into the flexor pollicis lungus and in 21 (36%) in the flexor digitorum profundus, a total of 58 insertions in 54 forearms, in four forearms, the muscle gave rise to two tendons. The Gantzer muscle giving rise to two tendon was also noticed by Shirali *et al*.^14^ in four. Limbs. Oh *et al*.^10^ also reported in one forearm that the Gantzer muscle originated two tendons. Jones *et al*.^13^ observed that in one of their cases the Gantzer muscle gave rise to three tendons, two of them inserted into the flexor pollicis lungus and the other in the flexor digitorum profundus. We did not register any case with more than two tendons.

Our findings are very close to those of Jones *et al*.^13^ who reported the insertion of the Gantzer muscle into the flexor pollicis longus muscle in 36 forearms and in the flexor digitorum profundus in 15 forearms (28.8%).

The classic Anatomy treaties such as Testut^2^ and Le Double^3^ reported that the Gantzer muscle receives innervation only from the anterior interosseous nerve, which was also recorded by Al Qattan,^8^ Hemmady *et al*.,^6^ Shirali *et al*.,^14^ Malhotra *et al*.,^12^ Ballesteros *et al*.,^9^ and Dellon and Mackinnon.^7^ We agree with these authors, because in all limbs the Gantzer muscle was innervated only by the anterior interosseous nerve. Jones *et al*.^13^ in 8% of 80 dissections reported that the Gantzer muscle received innervation of the median nerve, besides receiving by the anterior interosseous nerve (double innervation). Mangini^5^ in 7%, and Kida^20^ also in 7% reported that the Gantzer muscle was innervated by the median nerve.

We observed that the anterior interosseous nerve was more frequently crossed by the muscular component of Gantzer of muscle in only nine limbs (16.6%) the anterior interosseous nerve was crossed by the tendon component, which is in agreement with Oh *et al*.^10^ who called this most common arrangement type A, and the one that the nerve was crossed by the tendon component type B. The author named type C when the Gantzer muscle had a parallel path in relation to the anterior interosseous nerve, a provision which we noticed in six forearms (11%).

It is controversial whether the Gantzer muscle can cause the anterior interosseous nerve syndrome or even the pronator teres syndrome. Ballesteros *et al*.^9^ reported that in the 12 forearms they analyzed, the Gantzer muscle was located on the ulnar side parallel to the anterior interosseous nerve, being very unlikely to be the cause of the anterior interosseous nerve syndrome. Lister^21^ and Spinner^22^ report that the Gantzer muscle can cause the anterior interosseous nerve syndrome, especially when the Gantzer muscle is hypertrophied, but they do not believe that it may cause compression of the median nerve. Shirali *et al*.^14^ disagree, because they stand that theoretically, in the three cases in which they registered the presence of the Gantzer muscle anterior to the median nerve, the latter can indeed cause nerve compression.

Al Qattan^8^ reports that the median nerve can be influenced by the Gantzer muscle in two situations: when the nerve passes posterior to the deep head of the pronator teres muscle or when the deep head of the pronator is missing. We recorded eight limbs in which the deep head of the pronator was absent, but in all these parts the Gantzer muscle passed posterior to the median nerve.

Kaplan and report Spinner^23^ report two situations in which the median nerve may undergo compression by the Gantzer muscle. First, when the Gantzer muscle inserts in the superficial flexor muscle near its arcade. Second, when the muscle is perforated by the median nerve. We agree that in these two situations nerve compression may occur. We did not record these anatomical variations in our cases.

Dellon and Mackinnon^7^ reported that the hypertrophied Gantzer muscle, even passing anterior the anterior interosseous nerve, may compress the nerve between the Gantzer muscle and the aponeurotic muscle structures of the pronator teres muscles and flexor digitorum superficialis. They report that in the most distal part of its course the nerve may cross posterior to the tendon of the Gantzer muscle, and thus compress the anterior interosseous nerve branch to the square pronator, causing weakness on forearm pronation. We were uncertain about this statement of Dellon and Mackinnon,^7^ since it was registered in 31 forearms dissected that the muscle is always situated posterior to the nerve as its tendon could pass anterior to the branch to the square pronator.

We affirm that the hypertrophied Gantzer muscle can cause the anterior interosseous nerve syndrome, but this was an uncommon finding in only six forearms (11%), all bilaterally we recorded the hypertrophied muscle.

Tabit *et al*.^24^ report the clinical case of a patient with incomplete anterior interosseous nerve syndrome, with paralysis only of the flexor pollicis lungus, which during surgery proved to be caused by the Gantzer muscle. Our findings show that in 26 cases, therefore almost 50%, the anterior interosseous nerve crossed only the branches to the flexor pollicis lungus or flexor digitorum profundus, in such circumstances it may cause incomplete anterior interosseous nerve syndrome.

Hill *et al*.^25^ reported 33 clinical cases of incomplete paralysis of the anterior interosseous nerve and that most of them regressed spontaneously, but they make no reference regarding the possibility of Gantzer of muscle causing these paralyzes.

## CONCLUSION

The Gantzer muscle, especially when hypertrophied and positioned anterior to the median nerve and to the anterior interosseous nerve may be the cause of nerve compression. Present in 68% of cases, in our view, the Gantzer muscle should be considered a normal anatomical pattern and not an anatomical variation.
